# B7-H3 is eligible for predicting clinical outcomes in lung adenocarcinoma patients treated with EGFR tyrosine kinase inhibitors

**DOI:** 10.1186/s12957-022-02634-x

**Published:** 2022-05-20

**Authors:** Ying Yang, Jun-feng Huang, Bing-qi Hu, Jing Zhou, Xian Wang, Zhen-zhong Feng, Yu-ting Chen, Fa-ming Pan, Huai-dong Cheng, Li-wen Chen

**Affiliations:** 1 Department of Laboratory Medicine, Second Hospital of Anhui Medical University, Hefei, China; 2 Department of Pathology, Second Hospital of Anhui Medical University, Hefei, China; 3 Department of Epidemiology and Biostatistics, School of Public Health, Anhui Medical University, Hefei, China; 4 Department of Oncology, Second Hospital of Anhui Medical University, Hefei, China

**Keywords:** *EGFR* mutation, Target therapy, Biomarkers, Survival, Prognosis

## Abstract

**Background:**

Not all lung adenocarcinoma (LUAD) patients with activating epidermal growth factor receptor (*EGFR*) mutations respond to tyrosine kinase inhibitors (TKIs) as intended. Thus, biomarkers are needed to identify patients who benefit most from EGFR-targeted therapy. Our previous in vitro data has shown that the co-signal molecule B7-H3 determines EGFR-TKI gefitinib susceptibility of *EGFR*-mutated LUAD cell lines, based on the potential crosslinking between B7-H3-induced signaling and EGFR signaling.

**Methods:**

We detected tumoral B7-H3 expression in the original biopsy from 56 treatment-naïve LUAD patients and analyzed the association between high/low B7-H3 expression with the clinical outcomes of first-line anti-EGFR therapy. The main criteria for the analysis of response were overall response rate (ORR), disease control rate (DCR), and progression-free survival (PFS), and the secondary criterion was overall survival (OS).

**Results:**

In the subgroups of B7-H3 high and low expression, the ORR were 16.0% (4/25) and 74.2% (23/31) (*p*<0.001), and the DCR were 36.0% (9/25) and 87.1% (27/31) (*p*<0.001), respectively. The PFS of B7-H3 high [median 8.7, 95% confidence interval (CI) 4.0–13.4] was significantly worse than that of B7-H3 low (median not reached) [HR 6.54 (95% CI 2.18–19.60), *p*=0.001]. The median OS was 15.9 (95% CI 10.0–21.8) months in the B7-H3 high cohort and 25.7 (95% CI 9.0–42.4) months in the B7-H3 low subjects [HR 2.08 (95% CI 1.07–4.02), *p*=0.03], respectively. Both the univariate and multivariate analyses identified B7-H3 as an independent factor associated with poor PFS (*p*=0.001, *p*=0.000) and OS (*p*=0.03, *p*=0.015).

**Conclusion:**

B7-H3 may serve as a potential biomarker to predict clinical outcomes in *EGFR*-mutated LUAD patients treated with first-line EGFR-TKIs.

**Supplementary Information:**

The online version contains supplementary material available at 10.1186/s12957-022-02634-x.

## Background

Of the non-small-cell lung cancer (NSCLC) patients, epidermal growth factor receptor (*EGFR*) mutations account for approximately 30% of cases in China [1], 12% in the USA [2], and 10% in France [3] and are most common among patients with lung adenocarcinoma (LUAD) [3, 4]. In-frame deletions in exon 19 (19 Del) and a point mutation in exon 21 that substitutes an arginine for a leucine at codon 858 (21 L858R) constitute nearly 90% of all *EGFR* mutations [5, 6]. Over the past decade, the tyrosine kinase inhibitors (TKIs) target EGFR has been demonstrated to improve clinical outcomes for NSCLC patients harboring activating *EGFR* mutations [7]. Unfortunately, approximately 30% of patients exhibit primary resistance to EGFR-TKIs [8, 9], and the factors involved in de novo resistance remain unidentified. Thus, more predictive clinical and biological characteristics are needed to identify patients who will benefit most from anti-EGFR therapy.

The co-signal molecule B7-H3 (CD276) is abnormally upregulated in NSCLC and plays a negative role in cancer progression [10–12]. Previous studies have indicated that tumoral B7-H3 triggers pro-tumorigenic signals to promote cancer invasion, migration, angiogenesis, drugs sensitivity, and Warburg effect in a series of solid tumors including NSCLC [13–20]. Our previous in vitro study has shown that *B7-H3* knock-out increased gefitinib susceptibility of LUAD cell lines harboring 19 Del or 21 L858R mutations. Our further results uncovered the potential crosslinking between B7-H3-induced signaling and EGFR signaling in *EGFR*-mutated LUAD cell lines [21]. Thus, we speculate that the level of B7-H3 expression in LUAD is associated with EGFR-TKIs response.

In this study, we retrospectively analyzed the association of stratified (high *vs.* low) B7-H3 expression with clinical outcomes of LUAD patients treated with the first-line EGFR-TKIs. The findings contribute to evaluate B7-H3 profiling as a potential biomarker to identify patients at the screening who may derive improved clinical benefit from EGFR-targeted therapy.

## Methods

### Patients

From October 2016 through May 2021, a total of 56 LUAD patients in the Second Hospital of Anhui Medical University were enrolled in this study. The inclusion criteria were as follows: (1) advanced (stage IIIB, IIIC, and IV) LUAD patients harboring activating *EGFR* mutations, including 19 Del and 21 L858R; (2) patients were treatment-naive and received gefitinib (250 mg, q.d), Icotinib (125 mg, t.i.d), or erlotinib (150 mg, q.d) until disease progression or the advent of intolerable adverse effects; and (3) duration of treatment was at least 3 months at the time of data analysis. The exclusion criteria were as follows: (1) patients with age ≤18 years old; (2) sufficient information on treatment was unavailable. All patients provided written informed consent for the collection and analyses of tissue samples. The study was conducted in the principles of the Declaration of Helsinki and was approved by the Ethics Committee for the Second Hospital of Anhui Medical University (NO. 2021SHAMU0014).

The main criteria for the analysis of response were overall response rate (ORR), disease control rate (DCR), and progression-free survival (PFS), and the secondary criterion was overall survival (OS). The above indicators were evaluated according to the classified tumor response and disease progression based on Response Evaluation Criteria in Solid Tumors (RECIST version 1.1) [22, 23].

### Assessment of *EGFR* mutations

Activating *EGFR* mutations, 19 Del and 21 L858R of 56 patients were analyzed by using fresh, frozen specimens obtained from the original biopsy before any treatment. Tissue DNA was isolated by the TIANamp Genomic DNA kit (Spin Column) (TIAGEN Biotech, Beijing, China). Exon 19 Del and 21 L858R were detected by ADx-AMRS (Amplified Refractory Mutation System) *EGFR* Mutations Detection Kit (Amoy Diagnostics, Xiamen, China) and MX3000P (Stratagene, La Jolla, USA) real-time PCR system according to the manufacturer’s instructions. A positive or negative result could be reached if it met the criterion that was defined by the manufacturer’s instruction. All the mutant results of ADx-AMRS were confirmed by direct DNA sequencing.

### Tissue processing and B7-H3 expression quantification

B7-H3 expression was analyzed by immunohistochemistry on formalin-fixed, paraffin-embedded (FFPE) slides. In brief, sections of 4-μm thickness were performed heat-mediated antigen retrieval with citrate buffer (10-mM citrate, 0.05% Tween 20, pH 6.0) for 20 min at 100 °C. The slides were then removed from heat and allowed to cool down at room temperature in the buffer for 20 min. Next, immunostaining was performed by using the anti-CD276 antibody [SP206] (ab227670) (Abcam, Cambridge, MA, USA) at a dilution of 1:100 for 10 min at room temperature. The slides were then incubated with Biotin-Streptavidin Horseradish Peroxidase (HRP) Detection System (SP-9000, ZSGB-BIO, Beijing, China) and 3,3N-diaminobenzidine tertrahydrochloride (DAB) (ZLI-9017, ZSGB-BIO). By using the Olympus BX51 Microscopes (Tokyo, Japan), B7-H3 expression was evaluated by two pathologists in a blinded manner to avoid unintentional bias. All procedures followed the manufacturers’ protocols.

**Fig. 1:**
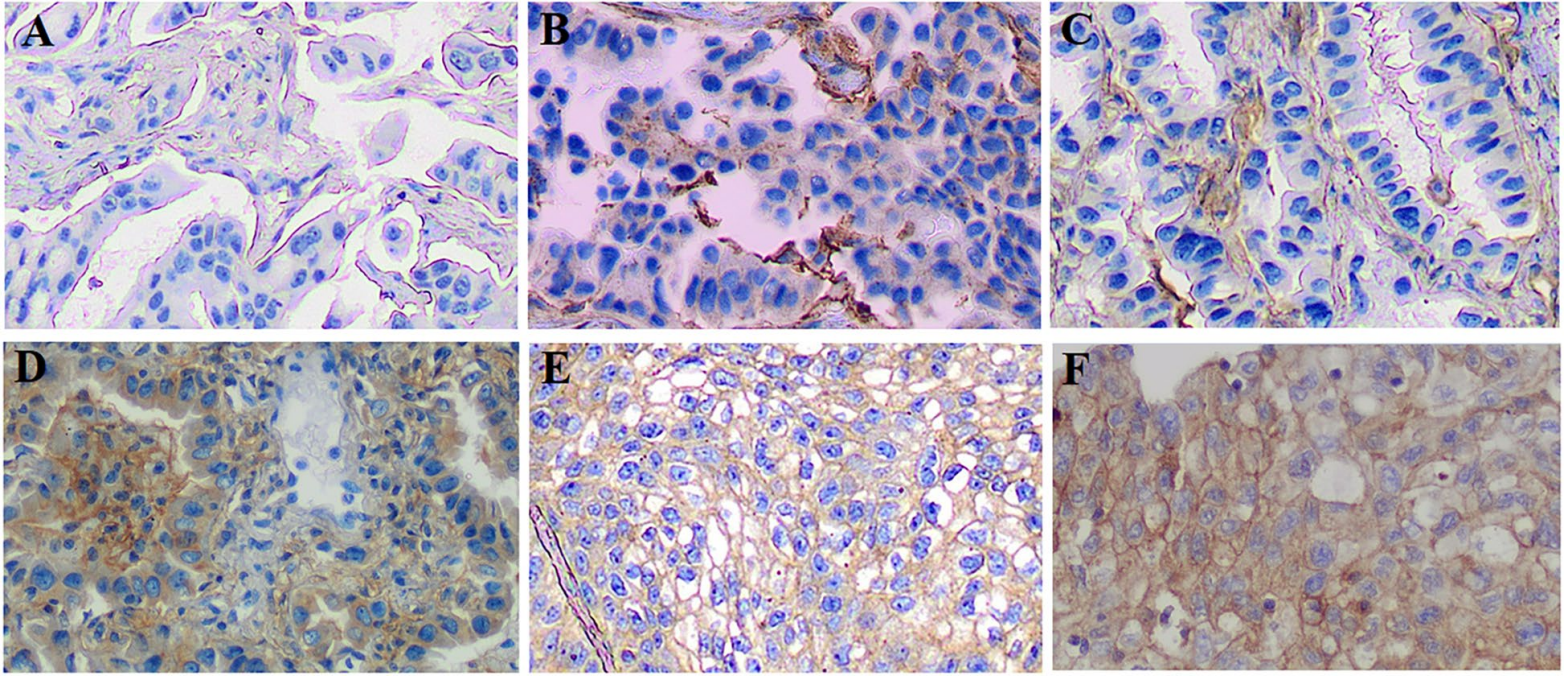
Immunohistochemical evaluation of tumoral B7-H3 expression in LUAD. **A** B7-H3 intensity 0 (negative). **B**, **C** B7-H3 low expression. **D–F** B7-H3 high expression (**D** cytoplasmatic staining. **E** Membranous staining. **F** Membranous and cytoplasmatic staining) (×200)

An immunohistochemical grading was made by incorporating the intensity of staining and the percentage of positive tumor cells as previously described [24–26]. The intensity of membranous and cytoplasmatic B7-H3 expression in lung cancer cells was defined traditionally as 0 (no staining), 1 (weak), 2 (intermediate), and 3 (strong). The percentage of positive cells was categorized as follows: 0 (<5%), 1 (5–25%), 2 (26–50%), 3 (51–75%), and 4 (>75%). An overall histochemical score was assigned to each case by multiplying the staining intensity by the percentage grade, which yielded a range from 0 to 12. All the specimens were divided into two groups: B7-H3 low (<6) and B7-H3 high group (6–12) (Fig. 1).

### Statistical analysis

Ratio comparison of high/low B7-H3 expression between groups defined by demographic and pathological characteristics (age, sex, tumor size, staging, mutation subtypes, and TKIs) and of ORR or DCR between patients with stratified B7-H3 expression was performed using Pearson’s chi-square (*χ*^2^) or Fisher’s exact test. Univariate and multivariate Cox proportional hazards models were fitted to assess the hazard rates (HRs, 95% CIs) of these demographic and pathological features on anti-EGFR response. The survival characteristics of B7-H3 high/low subgroups were analyzed and plotted to visualize by the Kaplan-Meier model (log-rank test). All analyses were performed using the SPSS version 22.0 (Chicago, IL). *P*<0.05 was considered statistically significant.

## Results

### Relationship between tumoral B7-H3 expression and clinicopathological characteristics

**Fig. 2:**
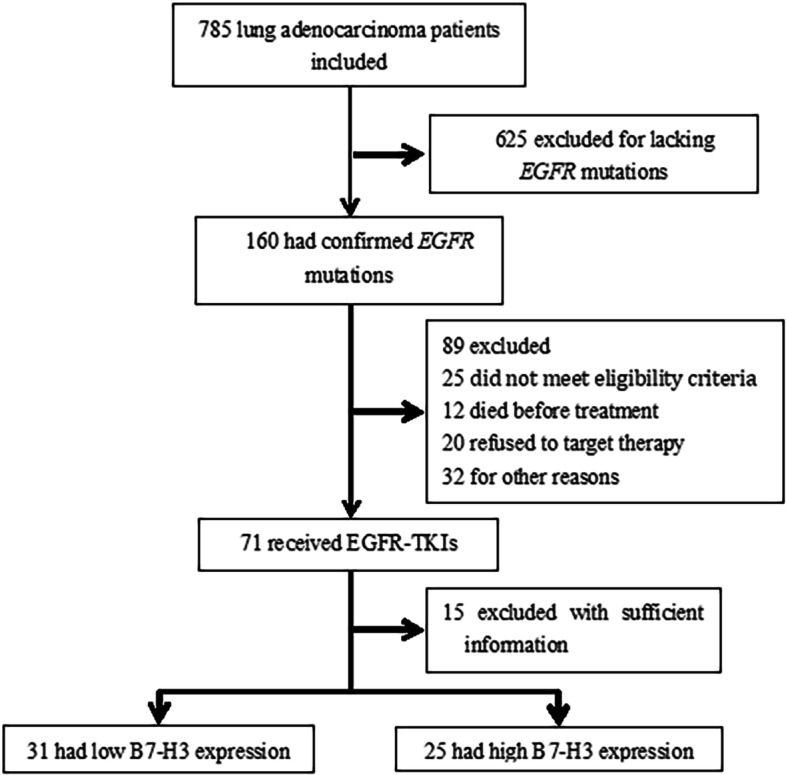
Trial profile at cutoff date for analysis (May 12, 2021)

Figure 2 shows the trial profile, and Table 1 shows the baseline characteristics of the 56 LUAD patients included in this study. The median age was 64 years, with a maximum age of 82 and a minimum of 32 years. There are no significant differences of the proportions of high/low B7-H3 expression between <60 (*n*=24) and ≥60 (*n*=32) aged, and between male (*n*=31) and female (*n*=25) patients. Most patients (*n*=54) did not have a smoking history except two former smokers (≥1 year since cessation). Also, B7-H3 expression levels were observed not to be significantly related to the clinicopathological characteristics including tumor size (≤30 mm vs. >30 mm) and staging (III B/C vs*.* IV). The association between B7-H3 expression and *EGFR* mutation patterns (19 Del and 21 L858R) was also assessed and again found no statistical difference. Of these patients, 30 received gefitinib, 22 received Icotinib, and 4 received erlotinib as the first-line therapy, respectively. Being a retrospective study, the participated patients are randomized to receive anti-EGFR therapy and the distribution of high/low B7-H3 expression was insignificant (*p*=0.839, Fisher’s exact test in R×C contingency tables). Taken together, the detection prior to EGFR-targeted therapy showed no significant differences of B7-H3 expression between age, sex, tumor size and staging, mutation patterns and selected EGFR-TKIs.

**Table 1:** Correlation between tumoral B7-H3 and clinicopathological features of LUAD patients

	*n* (%)	B7-H3 expression		*P*
		low	high	
Age (years)				*0.485*
<60	24 (42.9%)	12 (38.7%)	12 (48.0%)	
≥60	32 (57.1%)	19 (61.3%)	13 (52.0%)	
Gender				*0.320*
Male	31 (55.4%)	19 (61.3%)	12 (48.0%)	
Female	25 (44.6%)	12 (38.7%)	13 (52.0%)	
Tumor size (mm)				*0.651*
≤30	22 (39.3%)	13 (41.9%)	9 (36.0%)	
>30	34 (60.7%)	18 (58.1%)	16 (64.0%)	
Pathological stage				*0.089*
III	16 (28.6%)	6 (19.4%)	10 (40.0%)	
IV	40 (71.4%)	25 (80.6%)	15 (60.0%)	
*EGFR* mutation				*0.931*
19 Del	31 (55.4%)	17 (54.8%)	14 (56.0%)	
21 L858R	25 (44.6%)	14 (45.2%)	11 (44.0%)	
EGFR-TKIs				*0.839*^¶^
Gefitinib	30 (53.6%)	16 (51.6%)	14 (56.0%)	
Icotinib	22 (39.3%)	12 (38.7%)	10 (40.0%)	
Erlotinib	4 (7.1%)	3 (9.7%)	1 (4.0%)	

^¶^Fisher’s exact test in RxC contingency tables

### EGFR-TKI response rates in LUAD patients with high and low B7-H3 expression

Thirty-six patients (64.3%) were still alive at the time of the study. The median follow-up time was 19.6 months, and the min to max follow-up time was 2.1–42.8 months. Of the 56 patients who could be evaluated, no patients had a complete response (CR), 27 had a partial response (PR), 9 had stable disease (SD), and 20 had progressive disease (PD). Accordingly, the total ORR and DCR are 48.2% (27/56) and 64.3% (36/56), respectively. The PR, SD, and PD numbers in B7-H3 high patients were 4, 5, and 16, and in the B7-H3 low cohort were 23, 4, and 4, respectively, Accordingly, in the subgroups of B7-H3 high and low, the ORR was 16.0% (4/25) and 74.2% (23/31) (*p*<0.001, chi-square test), and the DCR were 36.0% (9/25) and 87.1% (27/31) (*p*<0.001, chi-square test), respectively.

### Survival analysis of anti-EGFR therapy associated with stratified B7-H3 expression

**Fig. 3:**
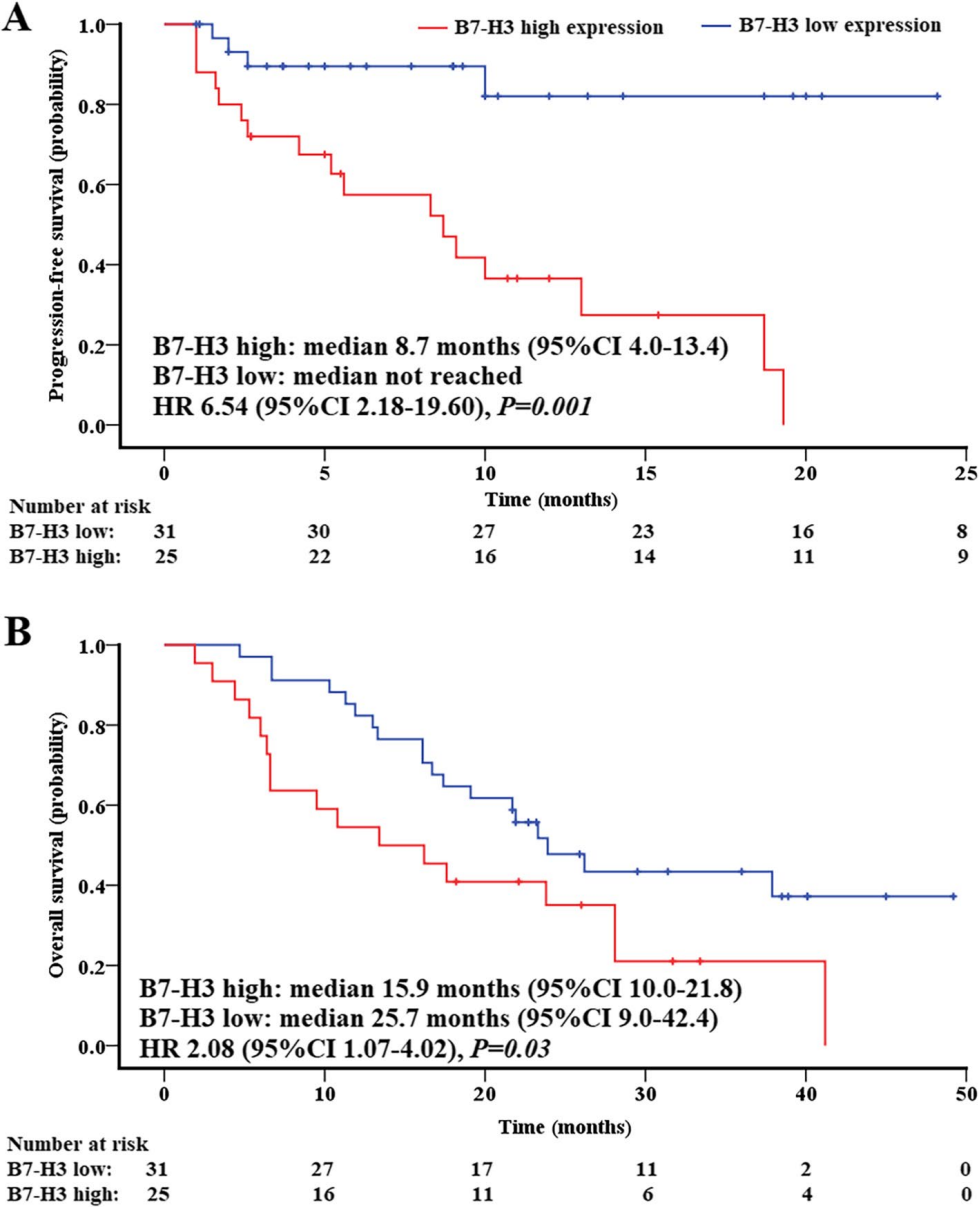
Kaplan-Meier survival curves of B7-H3 low/high LUAD patients treated with first-line EGFR-TKIs. **A** Progression-free survival. **B** Overall survival

Patients with B7-H3 high expression had significantly worse PFS [median 8.7, 95% CI 4.0–13.4] than that of B7-H3 low (median not reached) [HR 6.54 (95% CI 2.18–19.60), *p*=0.001 by the stratified log-rank test] (Fig. 3A). The median OS was 15.9 (95% CI 10.0–21.8) months in the B7-H3 high group and 25.7 (95% CI 9.0–42.4) months in the low B7-H3 subjects [HR 2.08 (95% CI 1.07–4.02), *p*=0.03 by the stratified log-rank test], respectively (Fig. 3B). Overall, the tumoral B7-H3 level is closely relevant to clinical outcomes of the first-line ant-EGFR therapy in LUAD.

Finally, both the univariate and multivariate analysis identified B7-H3 as an independent factor associated with poor PFS (*p*=0.001, *p*=0.000) and OS (*p*=0.03, *p*=0.015) in LUAD patients receiving anti-EGFR therapy. However, the clinicopathologic variables including age, sex, tumor size, EGFR-TKIs, and mutation subtypes have no correlation with poor survival (supplementary Table 1). The smoking history of patients was not incorporated in this study since only 2 patients were categorized as former smokers (≥1 year since cessation). Taken together, these results suggested that B7-H3 is eligible for acting as a predictor in LUAD patients treated with first-line EGFR-TKIs.

## Discussion

Anti-EGFR-targeted therapy has brought many benefits to *EGFR*-mutated NSCLC patients [27]. Previous study based on meta-analysis has shown that PD-L1 expression might be a predictive biomarker for *EGFR*-mutant NSCLC treated with EGFR-TKIs [28]. In this study, we showed that the expression level of tumoral B7-H3 is eligible for precise prediction of EGFR-target therapy in *EGFR*-mutated LUAD patients. These results confirm our previous in vitro observation that *B7-H3* knock-out increased gefitinib susceptibility of LUAD cell lines harboring exon 19 Del and 21 L858R mutations [21]. B7-H3 effects may be derived from the underlying cross-linking between EGFR signaling and B7-H3-induced signaling which share the majority of downstream cascades in tumor cells [15–20, 29]. This facilitates the interaction between the two molecules, as has been proved by our results [21], through the mutual regulation of the potentially constructed signaling network. Thus, it is very likely that B7-H3-induced signaling acts as alternative activating pathways for overcoming EGFR-TKI effects.

Of the 56 LUAD patients treated with the first-line EGFR-TKIs, the B7-H3 low cohort had significantly higher ORR and DCR and better PFS and OS than patients with B7-H3 high. Several studies have also examined the prognostic value of B7-H3 in NSCLC patients. Altan et al. have shown that only a high B7-H3 level (highest 10% vs*.* lowest 90%) was associated with poor OS while no correlation with survival was observed in B7-H3 positive vs*.* negative cases [11]. On the other hand, Inamura et al. showed that high B7-H3 expression was associated with shorter lung cancer-specific survival and OS in moderate/heavy-smoking patients (smoking index, SI ≥ 400) but not in non/light smoking patients (SI < 400) [10]. These results indicated that the association of B7-H3 expression with OS is group-limited. Whether there is a corresponding relationship between the top 10 percentile and the moderate/heavy-smoking patients is yet to be confirmed, although the two studies have unanimously shown the consistent association of elevated B7-H3 with smoking history. Our study differs from these studies in that (1) the smoking history was not incorporated since only 2 patients were categorized as former smokers (≥1 year since cessation), (2) we compared both the short-term (ORR, DCR, PFS) and long-term (OS) indicators in LUAD patients defined as B7-H3 high and low, and (3) we take treatment measure, the anti-EGFR therapy into B7-H3-based survival observation. Overall, our study demonstrates that B7-H3 acts as a predictor of clinical outcomes in LUAD patients treated with EGFR-TKIs.

It is to be noted that total ORR (48.2%) of the 56 patients in this study is substantially lower than previous clinical trials including First-SIGNAL (55.4%) [30], EURTAC (58–64%) [31], WJTOG3405 (62.1%) [32], IPASS (71.2%) [33], NEJ002 (73.7%) [9], and OPTIMAL (83%) [34], all of which were likewise based on first generation EGFR-TKI treatment. One possible explanation is the fact that this is a retrospective study. In most cases, the available medical data were gathered from repeated hospitalization, which may lead to the loss of the follow-up of patients with rather stable diseases [35]. As a result, no patients were observed to have a complete response in our study. Another possibility is the limitations of the small sample size of this study, thus further validation in a larger patient cohort is needed. On the other hand, DCR, a composite of ORR and SD, reached up to 87.1% in the B7-H3 low cohort, further indicating the predictive role of B7-H3 in anti-EGFR therapy since DCR is useful to measure the efficacy of therapies that have tumoristatic effects rather than tumoricidal effects [23]. Our results showed that the distribution of high and low B7-H3 expression has no association with age, sex, tumor size, staging, and *EGFR* mutation patterns. Similar results have been demonstrated in a study using samples from 3 retrospective cohorts of NSCLC patients [11]. However, baseline data from other groups showed that tumoral B7-H3 expression was higher in males, smokers, and more frequent in NSCLC patients with poor differentiation, larger tumor size, and wild-type *EGFR* [10, 12]. The inconsistency may in part resulted from the sampling variation as the patients in the present study all had advanced (III B/C and IV stage) diseases, with *EGFR* mutations and almost all had never smoked. It is unclear if *EGFR* mutations down-regulate B7-H3 expression to favor tumor immune escape in this context. Thus, a prognostic risk model, as it was generated in lung squamous cell cancer analysis, will be conducive to further elucidate the association between the two genes [36]. In our study, both the univariate and multivariate analysis showed that B7-H3 is an independent factor associated with poor PFS and OS whereas the clinicopathologic variables (age, sex, tumor size, TKIs, and mutation subtypes) have no correlation with poor survival of anti-EGFR therapy. These results contrast with previous studies which indicated a better response was associated with 19 Del rather than 21 L858R mutations [8, 37], and an age between 61 and 70 years of 197 patients received erlotinib [8]. However, the West Japan Oncology Group (WJTOG) 3405 study reported no associations between PFS and sex, age (<65 vs*.* ≥65 years), smoking history, and mutation subtypes in NSCLC patients, irrespective of gefitinib or cisplatin plus docetaxel treatment [32]. The possible explanations for these inconsistencies are differences in age grouping and of EGFR-TKIs used between studies. Further study using the propensity score matching analysis may be needed [38, 39].

## Conclusions

In conclusion, our study demonstrates that higher tumoral B7-H3 expression is correlated with poorer response in LUAD patients treated with EGFR-TKIs. Thus, B7-H3 is eligible for acting as a predictor for anti-EGFR therapy in *EGFR*-mutated LUAD. Together, this work furthers our understanding of the EGFR targeted therapy in LUAD by proactively identification of patients who will benefit most from anti-EGFR treatment.

## Supplementary Information

**Additional file 1: Additional file Table S1.** Univariate and multivariate Cox proportional hazards regression models for PFS and OS.

## Data Availability

All data generated or analyzed during this study are included in this published article and its supplementary information files.
