# Supplementary material for: B7-H3 is eligible for predicting clinical outcomes in lung adenocarcinoma patients treated with EGFR tyrosine kinase inhibitors
Source: World J Surg Oncol. 2022 May 20;20:159. doi: 10.1186/s12957-022-02634-x (PMC9121599; doi:10.1186/s12957-022-02634-x)
Supplement: Supplementary file 1 — Additional file 1: Additional file Table S1. Univariate and multivariate Cox proportional hazards regression models for PFS and OS. [file 12957_2022_2634_MOESM1_ESM.docx]

Supplementary Table 1 Univariate and multivariate Cox proportional hazards regression models for PFS and OS.

| Variables | | PFS | | | | | | OS | | | | | |
| --- | --- | --- | --- | --- | --- | --- | --- | --- | --- | --- | --- | --- | --- |
|  |  | Univariable analysis | | | Multivariable analysis | | | Univariable analysis | | | Multivariable analysis | | |
|  |  | OR (95% CI) | | *P* | OR (95% CI) | | *P* | OR (95% CI) | | *P* | OR (95% CI) | | *P* |
| Age (years) | ≥ 60 (*vs.* <60) | 0.952  (0.394-2.305) | *0.914* | | 1.400  (0.360-5.450) | *0.627* | | 1.373  (0.686-2.749) | *0.371* | | 1.238  (0.432-3.544) | *0.691* | |
| Gender | Male (*vs.* Female) | 0.651  (0.269-1.576) | *0.341* | | 0.593  (0.160-2.201) | *0.435* | | 0.776  (0.386-1.561) | *0.478* | | 1.173  (0.387-3.555) | *0.778* | |
| B7-H3 | Low (*vs.* High) | 6.540 (2.182-19.604) | *0.001* | | 7.816 (2.507-24.372) | *0.000* | | 2.076 (1.072-4.021) | *0.03* | | 2.315 (1.173-4.568) | *0.015* | |
| Tumor size (mm) | >30 (*vs.* ≤30) | 2.846  (0.971-8.346) | *0.057* | | 2.466  (0.410-14.821) | *0.324* | | 1.997  (0.900-4.433) | *0.089* | | 2.820  (0.832-9.556) | *0.096* | |
| Pathological stage | IV (*vs.* III) | 0.808  (0.306-2.134) | *0.667* | | 0.505  (0.073-3.510) | *0.490* | | 0.843  (0.408-1.743) | *0.645* | | 1.316  (0.376-4.607) | *0.667* | |
| EGFR mutation | 19 Del (*vs.* 21 L858R) | 2.577  (0.684-9.706) | *0.162* | | 2.399  (0.403-14.283) | *0.337* | | 1.884  (0.733-4.840) | *0.188* | | 1.569  (0.509-4.829) | *0.433* | |
| EGFR-  TKIs^ξ^ | Gefitinib *vs.* Icotinib | 1.541  (0.634-.3.749) | *0.340* | | 3.688  (0.722-18.831) | *0.117* | | 1.223  (0.615-2.430) | *1.223* | | 2.038  (0.628-6.614) | *0.236* | |

^ξ^Erlotinib is not analyzed in the models due to small sample size.
